# Supplementary material for: Learning curve and surgical outcome of robotic assisted colorectal surgery with ERAS program
Source: Sci Rep. 2022 Nov 29;12:20566. doi: 10.1038/s41598-022-24665-w (PMC9709162; doi:10.1038/s41598-022-24665-w)
Supplement: Supplementary file 2 — Supplementary Information 2. [file 41598_2022_24665_MOESM2_ESM.zip › RAL Raw data-Ñ[▒K/066/HS_ñw╝╨░O▒Kñσ.pdf]

正式摘要(電子簽章：000000213922353)

W52 / W52 ☐ 英文標題(注意:無英文姓名-請補病人基本檔!) ☐【讀取】憑證

臺中榮民總醫院
出院病歷摘要

轉入號： 轉出號：

[redacted]

(8)流水編號	
(9)入院日期	110/03/24 (2021) 直腸外科 W52 - 121
(10)轉科日期	
(11)出院日期	110/04/07 (2021) 住院天數計 14 日

診斷

- 入院：1. S-colon Ca
- 出院：1. S-colon Ca, Adenocarcinoma, moderately differentiated, arising in a tubulovillous adenoma, pT2N1a, stage III.
  - s/p robotic AR on 2021/03/25

主訴

- bloody stool for 1 month

病史

- This 65 y/o female with a hx of chronic constipation under probiotics for 20+ yrs.Her ADL was totally independent.
  This time, she complained of bloody stool about 1 month ago. There was no change of defecation habits, body weight loss, nor general weakness. She first went to 光田H for help. Abd CT showed sigmoid tumor about 4.1 cm with colonic wall invasion and suspected LN involvement. LGI scope was done and showed: Tumor, colon, 30 cm from anal verge, colonoscopic biopsy -Adenocarcinoma, moderately differentiated. Under the impression of sigmoid colon cancer, she was admitted for Robotic AR and ERAS protocol.

體檢發現

-
  - General appearance :
    - easy looking , conscious clear , without cardiopulmonary distress
    - Vital signs自動帶入date 體溫(BT) 脈搏(P) 呼吸(R) 血壓(BP) 身高(BH) 體重(BW) BMI
      20210324 36C 80/min 20/min 140/85mmHg 159cm 55.4kg 21.9
    - Vital signs: B.P. 140/85 mmHg , P.R. 80 /min , R.R. 20 /min , B.T. 36 ℃
  - HEENT :
    - Eyes: no anemic conjunctiva, no icteric sclera, normal EOM
    - ENT: no otorrhea, no rhinorrhea, no erythematous mucosa, no injected tonsil, no deformity
  - Neck: supple, freely movable, trachea midline,no deformity, no engorged jugular vein , no palpable thyroid or lymph node enlargement
  - Chest:
    - Inspection: Grossly normal, symmetric and fully expansion, no accessory muscle used, no operation scar, no spider angiomata, no gynecomastia
    - Palpation: no palpable mass or nodule, no subcutaneous emphysema, no tenderness
    - Ausculation: clear breathing sound
  - Heart:
    - Palpation: no thrill, no heave
    - Percussion: normal heart size
    - Ausculation: regular heart beat, normal S1, S2, no gallop, no murmur
  - Extremities: No deformity, full ranged of motion without limitation, no clubbing finger or nail cyanosis, no pitting edema, no ulcer or pigmentation, no obvious skin lesion, no varicose vein, no bone, joint or muscle tenderness

- **CRS CONDITION**
  - Karnofsky performance status: (100) Normal, no complaints, no evidence of disease
  - PE of Abdomen:
  - CEA: 6.36 ng/ml
  - Imaging Studies:
  - Colonoscope report: S-colon tumor, 30 cm from anal verge
  - Figures:
    1. colon & rectum

手術日期及方法

- 手術日期及方法20210325 Robot Assisted Colectomy(L)
  20210406 Chemo-port
- 手術結果及發現：
  - 1. One 4x5 cm tumor located at distal S-colon 2. There was one liver cyst noted, no visible peritoneal seeding.

住院治療經過

- After admission, robotic AR was done smoothly on 2021/03/25. On ERAS protocol, she tried BLAND II diet on 03/27 without abdomen discomfort. Neck pain was mentioned on 03/29, hydration and pain cotrol were givan. CVC was removed on 03/30. Pathological report showed S-colon, Adenocarcinoma, moderately differentiated, arising in a tubulovillous adenoma, pT2N1a, stage IIIA. After discussing with her, adjuvant chemotherapy would be performed on 4/30 and chemoport implantation was done on 4/6. Under the stable condition, she was discharged and would be followed-up at OPD.

合併症

- nil

一般檢查

- 累積報告：

DATE	TIME	ALB	TP	BIL.T	BIL.D	ALKP	AST	ALT	LDH	CHOL	TG	HDL-C	LDL	UA	NH3	AMY
1100324	1533	4.4	7.4	0.70	0.13	117	42	59	165	271	155	82		3.8		
1100324	1549												162			
1100325	1233	3.4				89	36	48								
1100329	0936	3.7	6.3	0.7	0.2	90	24	28	155							

DATE	TIME	WBC	RBC	HGB	HCT	MCV	PLT	BLST	NEUT	BAND	SEG	LYM	MON	EOS	BAS
1100324	1528	8030	4.29	14.3	41.9	97.7	404		57.6			34.1	7.2	0.6	0.5
1100325	1233	7980	3.80	12.3	36.0	94.7	332		79.7			18.8	1.4	0.0	0.1
1100329	0917	5850	3.94	12.8	37.7	95.7	355		60.6			28.9	6.8	3.4	0.3

DATE	TIME	NA	K	CL	CA	BUN	CREAT	Mg	P	CRP	ESR	PCT	LIPASE	LACTATE
1100324	1533	140	4.0	101	9.3	13	0.87			0.667				
1100325	1233	137	4.2	103	8.3	9	0.72							
1100329	0936	136	3.9	100	8.3	11	0.66							

DATE	TIME	GLUCOSE	PAB	TRF	ZN	CU	KETONE	CK	CKMB	PT-P	PT-C	APTT-P	APTT-C	TRO-T
1100325	1233	155												

特殊檢查

病理報告

- 活體組織切片病理報告　手術日期：**110/03/25**　病理號：**S1009884**

診斷醫師:吳致瑩 報告日期:110/03/31
Pathologic diagnosis:
1. Sigmoid colon, robotic anterior resection --- Adenocarcinoma, moderately differentiated, arising in a tubulovillous adenoma. Tumor invaded to muscularis propria, and metastasized to mesocolic lymph nodes (1/24). The bilateral cut ends are free of tumor.
2. IMA root, dissection --- Unremarkable fibroadipose tissue and lymph nodes (0/7).
3. Colon, cut end, distal, resection --- Melanosis coli.
Ancillary study for diagnosis:

- IHC stains for MLH1, MSH2, PMS2 and MSH6 are done on section E. All above markers are preserved.
  Prognostic and predictive factor:
  1. Tumor size: 5.5x3.5 cm.
  2. Depth of invasion: Muscularis propria.
  3. Histologic grade: G2: Moderately differentiated.
  4. Angiolymphatic invasion: Not identified.
  5. Perineural invasion: Present.
  6. Tumor deposits (discontinuous extramural extension): Not identified.
  7. Macroscopic tumor perforation: Not identified.
  8. Surgical Margins:
  a) Proximal Margin: Uninvolved by invasive carcinoma, distance of tumor from margin: 5.5 cm.
  b) Distal Margin: Uninvolved by invasive carcinoma, distance of tumor from margin: 2.8 cm.
  c) Radial or Mesenteric Margin: Uninvolved by invasive carcinoma, distance of tumor from margin: 7 cm.
  9. Lymph node status: Involved (1/31).
  10. Extranodal involvement: Not identified.
  11. Tumor regression grading S/P CCRT: No known presurgical therapy.
  12. Type of polyp in which invasive carcinoma arose: Tubulovillous adenoma.
  13. Additional pathologic findings: Not identified.
  14. Pathological TNM Stage: pT2N1a(According to the eighth edition, American Joint Committee on Cancer Staging Guidelines for Tumors).
  15. TNM descriptors: Not applicable.
  Gross description:
  The specimen consists of 1) a segment of colon, 12.2 cm in length and 4.5 cm in proximal cut end circumference, 6 cm in distal circumference, and mesocolic soft tissue. A gray tan firm annular elevated and centrally ulcerative tumor, 5.5x3.5 cm, is noted, 2.8 cm to the distal cut end. On cross section, the tumor invades to the muscular layer with 70 mm to the radial margin. The both cut ends are free of tumor grossly. 2) IMA root, a piece of tan yellow soft tissue, 2.8x1.8x0.5 cm. 3) distal cut end, a piece of tan mucosa-covered soft tissue, 3x2.1x0.8 cm. Representative part for section: A) proximal cut end B) distal cut end C-E) tumor F) radial margin G) all of specimen 2 H) specimen 3 J-M) lymph nodes. Blue ink indicated serosa; yellow ink indicated radial margin.
  Note:
  1. The interpretation of MMR panel and recommended studies are as below:
  (1) No loss of nuclear expression of MMR proteins: low probability of microsatellite instability-high (MSI-H).
  (2) Loss of nuclear expression of MLH1 and PMS2: testing for methylation of the MLH1 promoter and/or mutation of BRAF is indicated (the presence of a BRAF V600E mutation and/or MLH1 methylation suggests that the tumor is sporadic and germline evaluation is probably not indicated; absence of both MLH1 methylation and of BRAF V600E mutation suggests the possibility of Lynch syndrome, and sequencing and/or large deletion/duplication testing of germline MLH1 may be indicated).
  (3) Loss of nuclear expression of MSH2 and MSH6: high probability of Lynch syndrome (sequencing and/or large deletion/duplication testing of germline MSH2 may be indicated, and, if negative, sequencing and/or large deletion/duplication testing of germline MSH6 may be indicated).
  (4) Loss of nuclear expression of MSH6 only: high probability of Lynch syndrome (sequencing and/or large deletion/duplication testing of germline MSH6 may be indicated).
  (5) Loss of nuclear expression of PMS2 only: high probability of Lynch syndrome (sequencing and/or large deletion/duplication testing of germline PMS2 may be indicated).
  (6) Please note: There are exceptions to the above IHC interpretations. These results should not be considered in isolation, and clinical correlation with genetic counseling is recommended to assess the need for germline testing.
  2. This case has been peer reviewed by two doctors.

- #T-59470_2 #M-82633_2 2 1105 M0000F
  #T-C4560_2 #M-81406_2 0 1105 000000

其它

出院時情況

- 3.改門診治療

出院指示

-

藥名	劑量	劑量單位	用法	頻次	數量	數量單位	現況	天數	單位
Tonec tab 100mg	1	TAB	PO	BIDPRN	6	TAB	TH	3	天
Norvasc tab 5mg	1	TAB	PO	QD	7	TAB	TH	7	天
Cividoid gel 20g		TUB	TOP	BID	0	TUB	TH	1	瓶

出院計劃

主治醫師_2062F 蔣鋒帆 / 住院醫師_2334J 陳勇先

正式摘要(電子簽章：**000000213922353)**

W52 / W52

doctorRank:B
